# The effect of two novel amino acid-coated magnetic nanoparticles on survival in vascular endothelial cells, bone marrow stromal cells, and macrophages

**DOI:** 10.1186/1556-276X-9-461

**Published:** 2014-09-03

**Authors:** Qinghua Wu, Ning Meng, Yanru Zhang, Lei Han, Le Su, Jing Zhao, Shangli Zhang, Yun Zhang, Baoxiang Zhao, Junying Miao

**Affiliations:** 1 Shandong Provincial Key laboratory of Animal Cells and Developmental Biology, School of Life Science, Shandong University, Jinan 250100, China; 2 Institute of Organic Chemistry, School of Chemistry and Chemical Engineering, Shandong University, Jinan 250100, China; 3 School of Biological Science and Biotechnology, University of Jinan, Jinan 250022, China; 4 The Key Laboratory of Cardiovascular Remodeling and Function Research, Chinese Ministry of Education and Chinese Ministry of Health, Shandong University Qilu Hospital, Jinan 250100, China

**Keywords:** Magnetic nanoparticles, HUVECs, BMSCs, Macrophages, Nanotoxicity

## Abstract

Magnetic nanoparticles (MNPs) have been popularly used in many fields. Recently, many kinds of MNPs are modified as new absorbents, which have attracted considerable attention and are promising to be applied in waste water. In our previous study, we synthesized two novel MNPs surface-coated with glycine or lysine, which could efficiently remove many anionic and cationic dyes under severe conditions. It should be considered that MNP residues in water may exert some side effects on human health. In the present study, we evaluated the potential nanotoxicity of MNPs in human endothelial cells, macrophages, and rat bone marrow stromal cells. The results showed that the two kinds of nanoparticles were consistently absorbed into the cell cytoplasm. The concentration of MNPs@Gly that could distinctly decrease survival was 15 μg/ml in human umbilical vascular endothelial cells (HUVECs) or bone marrow stromal cells (BMSCs) and 10 μg/ml in macrophages. While the concentration of MNPs@Lys that obviously reduced viability was 15 μg/ml in HUVECs or macrophages and 50 μg/ml in BMSCs. Furthermore, cell nucleus staining and cell integrity assay indicated that the nanoparticles induced cell apoptosis, but not necrosis even at a high concentration. Altogether, these data suggest that the amino acid-coated magnetic nanoparticles exert relatively high cytotoxicity. By contrast, lysine-coated magnetic nanoparticles are more secure than glycine-coated magnetic nanoparticles.

## Background

Nanoparticles have been used for many fields because of their diversiform properties; meanwhile, growing concerns for their detrimental effects on human health have been taken to the agenda [1]. Nanoparticles based on iron oxide core (so-called magnetic nanoparticles (MNPs)) have been widely used in magnetic resonance imaging (MRI) [2-4], drug delivery devices [5], and environmental pollutant absorbents [6-9] for their superparamagnetic properties, smaller size but large surface-to-volume ratio, and increased reactivity. Emerging evidence has shown that nanotoxicity to biological system is scale-dependent, especially in particles below 20-nm diameter [10]. However, the bare nanoparticles are often coated with kinds of organic or other biological targeted materials, such as dextran [11-13], which may greatly influence their toxicity on multicellular organisms, increasing the complexity for toxicology evaluation. The key challenge we are facing is to find an effective approach to design surface-coated nanoparticles with enhanced functions as well as high biocompatibility.

Increasing studies report that the materials surface-coated with MNPs were intravenously administrated to diagnose cardiovascular diseases (e.g., atherosclerosis) and used for cancer therapy [6,11,14-17]. Once nanoparticles were administrated into the organism, they will enter through the blood vessel wall and then participate in blood circulation. Vascular endothelial cells lining the blood vessel, as the first line of defense, contact with the particles directly, which makes it considerably important to determine the nanotoxicity on vascular endothelial cells [18].

MNPs coated with kinds of biological materials have also been used as carriers for cell labelling [19,20]. Bone marrow stromal cells (BMSCs), as very important cells in the biological organism, act as sources of blood and tissue reproduction and are extensively used in cell therapy [21-24]. In the field of cell therapy, BMSC-derived cells are marked with magnetic nanoparticles so as to real-time resonance checking cells, which has got broad prospects [25]. However, the fact that many magnetic nanoparticles have the potential cytotoxicity on BMSCs makes a major obstacle for their successful application. So understanding the toxicity of the novel amino acid-coated magnetic nanoparticles on BMSCs is highly necessary.

To date, several kinds of MNPs have also been approved for disease detection by intraperitoneal injection [26,27] as well as intravenous injection [28]. Therefore, there is a wide range of interaction between macrophages and nanoparticles, which results in macrophage activation and causes subsequent inflammatory responses. Recent studies hint that MNP-labeled macrophages can be used for monitoring disease activity [29]. However, we still have no idea about what concentration of nanoparticles causes injury in macrophages. The human monocyte cell line THP-1 can differentiate into macrophages after phorbol-12-myristate acetate (PMA) stimulation, which is regarded as a widely used model to study macrophage function [30,31]. Therefore, in this study, we evaluated the cytotoxicity of nanoparticles on THP-1-derived macrophages.

Our previous studies showed that the MNP surface-coated with lysine or glycine could efficiently remove several kinds of anionic and cationic dyes under severe conditions [8,9], so the two kinds of nanoparticles are highly promising to be used in wastewater. However, we do not know whether the residue in water may have detrimental effects on human and animal cells.

In the present study, we hypothesized that the amino acid-coated MNPs might display some nanotoxicity in human important cells, including vascular endothelial cells and macrophages, and rat BMSCs. We made a study on the nanotoxicity in the three kinds of cells: human umbilical vascular endothelial cell (HUVECs), BMSCs, and THP-1-derived macrophages. The goal of this study was to detect the possible safe concentration of MNPs (average diameter = 10 nm) on the cells. We examined the nanotoxicity of the two kinds of MNPs by assessing their effects on cell morphological changes, viability, and apoptosis. These data suggest that the amino acid-coated magnetic nanoparticles exert relatively high cytotoxicity; by contrast, lysine-coated magnetic nanoparticles are more secure than glycine-coated magnetic nanoparticles.

## Methods

### Preparation and characterization of two novel amino acid-coated magnetic nanoparticles

Two kinds of Fe_3_O_4_ nanoparticles surface-coated with glycine (MNPs@GLy) and lysine (MNPs@Lys) were used in our experiments. The scheme for the synthesis of the nanoparticles and the characterization data such as transmission electronic microscope (TEM) images, the structures, and Fourier transform infrared spectroscopy measurements were determined as previously described by Zhang et al. [8,9]. The hydrodynamic size, polydispersity index, and zeta potential were detected by dynamic light scattering (DLS) (Malvern Zetasizer Nano ZS90, Malvern Instruments, Malern, UK). Since the bare Fe_3_O_4_ were originally coated with 3-glycidoxypropyltrimethoxysilane (GPTMS) and then were combined with glycine or lysine through chemical bonds, it means that high temperature cannot break the structure of MNPs. Thus, for our experiments, the nanoparticles were first sterilized at 121°C for 30 min and then were suspended in M199 medium (for HUVEC culture), Dulbecco's modified eagle's medium (DMEM) (Gibco, Carlsbad, CA, USA) medium (for BMSC culture) or RPMI1640 medium (for THP-1 culture). Stock solutions (concentration of 2 mg/ml) were sonicated for 10 min so as to break up the aggregates. Before each experiment, the nanoparticles were sonicated again for 5 min and blown top and down 10 times to achieve good suspension.

### Cell culture

HUVECs were cultured as described by Jaffe et al. [32]. The cells were remained in M199 medium (Gibco), supplemented with 15% fetal bovine serum (FBS) (Hyclone Lab Inc., Logan, UT, USA) and 10 IU/ml FGF-2, and incubated at 37°C incubation in humidified air with 5% CO_2_. Experiments were performed on the cells from 10 to 20 passages.

BMSCs were isolated and cultured as described previously [33]. Briefly, the BMSCs were isolated from femur and tibia marrows of 30-day-old neonatal male Wistar rats; the isolated cells were cultured in DMEM (low glucose), supplemented with 15% FBS (Hyclone Lab Inc.) and 10 IU/ml FGF-2, and remained at 37°C in humidified air with 5% CO_2_ incubator. Cells from 5 to 15 passages were used in our experiments.

Both HUVECs and BMSCs were exposed to MNPs after they were cultured for 24 h at the initial density of 1.5 × 10^4^/cm^2^. THP-1 cells were cultured in RPMI 1640 medium (Gibco) and supplemented with 10% FBS (Hyclone Lab Inc.) and 1% penicillin-streptomycin at 37°C in humidified air with 5% CO_2_. For the experiments, the cells were seeded at the density of 4 × 10^4^ cells/cm^2^ in 24-well plate and were stimulated with 100 ng/ml PMA (Sigma-Aldrich, St, Louis, MO, USA) for 96 h so as to be sufficiently differentiated into macrophages.

### Prussian blue staining assay

To visualize the bio-distribution of MNPs in cells, Prussian blue staining was performed according to manufacturer instruction. After cells were seeded in 24-well plate for 24 h, the cells were incubated with two kinds of MNPs (10 μg/ml) for 24 h. Then, the cells were fixed with 4% paraformaldehyde for 10 min, washed, and incubated for 30 min with potassium ferric-ferrocyanide (reagent for staining; Sigma). The cells were washed with running water for 5 min, then were counterstained with nuclear fast red. The pictures were taken under light microscopy (Olympus, Nikon, Tokyo, Japan).

### Cell viability assay

Cell viability was determined by using cell counting-8 (WST-8) kit (Sigma Chemical Co.). The detailed methods were in compliance with manufacturer instruction. Briefly, the cells were seeded in 96-well plate. The cells were treated with nanoparticles for 44 h before 10 μl of WST-8 solution was added. After being blended in a micro-oscillator, the cells were incubated for 4 h in 37°C incubator. In order to eliminate the interference of nanoparticles on absorbance value, the aggregated particles were removed by centrifugation at 1,500 rpm for 10 min. The OD values were observed at 450 nm by microplate reader (Tecan, San Jose, CA, USA).

### Hoechst 33258 staining

Briefly, the cells were fixed with 4% paraformaldehyde for 10 min after being washed with 1× PBS twice, then incubated with Hoechst 33258 (Sigma) at a final concentration of 10 μg/ml for 15 min at 37°C incubator. After the cells were washed with 1× PBS twice, digital images were taken under a fluorescence microscope (Nikon TE2000-U).

### Acridine orange staining

The cells were stained with acridine orange (AO; 5 μg/ml) (Fluka Chemical Corp., St. Louis, MO, USA) for 2 min at room temperature, then washed with 1× PBS twice so as to eliminate background interference. Dead cells characterized with nuclear concentration and fragmentation was observed under a laser scanning confocal microscope (Leica Microsystems, Wetzlar, Germany).

### Acridine orange (AO) and ethidium bromide (EB) double staining for THP-1-derived macrophages

Cell death (apoptosis or necrosis) was analyzed by measuring the permeability of cell membrane with AO-EB staining assay [34]. Because live cells are permeable to AO but not to EB and stained green, and dead cells are permeable to both AO and EB, and EB stained DNA red, it is easier to determine apoptotic cells or necrotic cells. Briefly, the cells were stained with cocktail of AO (100 μg/ml) and EB (100 μg/ml) in RPMI 1640 medium at 37°C for 10 min, then the cells were washed with PBS twice. Digital images were taken with Olympus inverted fluorescence microscope (Nikon TE2000-U).

### Cell necrosis assay

Cell necrosis was determined by measuring the release of lactic dehydrogenase (LDH); the assay was performed using LDH kit (Nanjing Jiancheng Chemical Industrial Co. Ltd., China) according to the manufacturer's instruction. The OD values were read at 440 nm using a microplate reader (Tecan).

### Statistical analysis

Data were expressed as mean ± SEM. The mean values were derived from at least three independent experiments. All statistical analyses were performed by using GraphPad Prism software with the method of one-way ANOVA (version 5; GraphPad Software Inc., San Diego, CA, USA). Differences at *P* < 0.05 were considered statistically significant.

## Results and discussion

### Physicochemical characteristics of two amino acid-coated magnetic nanoparticles

In this study, we investigated the MNPs coated with two kinds of amino acids (Gly or Lys). The physicochemical information of the nanoparticles was listed in Additional file 1: Table S1. In order to describe the hydrodynamic size, dispersity, and the surface charge of nanoparticles, we carried out the tests by DLS in different media and concentrations. The glycine-coated MNPs at a low concentration exert a mean hydrodynamic size of about 200 nm in water and M199 culture but have an increase of size at a high concentration. Similarly, the lysine-coated MNPs have a mean hydrodynamic size of about 280 nm in water, about 200 nm at a low concentration, while about 340 nm at a high concentration in M199 culture. Both particles remained a relatively good dispersity and negative charge.

**Figure 1:**
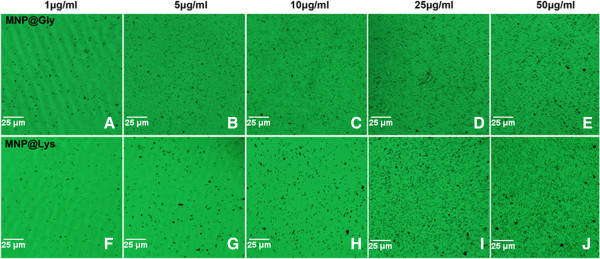
**The dispersion of two kinds of MNPs in the culture medium.** DMEM-L supplemented with 15% FBS incubated with nanoparticles at different concentrations. **(A-E)** MNPs@GLy and **(F-J)** MNPs@Lys.

Since the cell culture contains a variety of proteins from serum, the nanoparticles may interact with kinds of protein through electrostatic interaction and adsorption effect. Possible aggregations of nanoparticles make it difficult to assess the nanotoxicity. In order to directly explain the dispersity of nanoparticles in culture medium, two of the nanoparticles at different concentrations were incubated with DMEM-L for 48 h; then, pictures were taken to observe the dispersion of nanoparticles. We found no obvious accumulation of nanoparticles (Figure 1). These results further indicate a good dispersity of nanoparticles.

**Figure 2:**
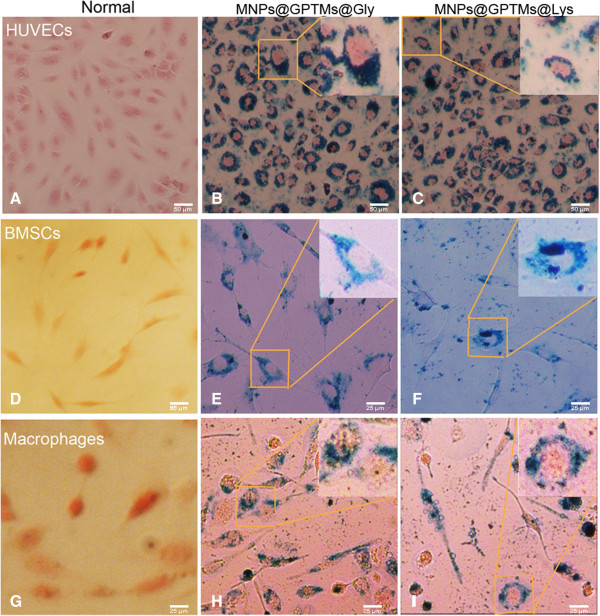
**Bio-distribution of two kinds of MNPs in cells.** The two kinds of amino acid-coated nanoparticles were absorbed in cells and dispersed in the cytoplasm. **(A-C)** HUVECs, **(D-F)** BMSCs, **(G-I)** macrophages. **(A, D, G)** Cells under normal conditions. **(B, E, H)** Cells were incubated with MNPs@Gly and **(C, F, I)** incubated with MNPs@Lys.

Supplementary physical or chemical information about MNPs@Lys and MNPs@Gly could be found in [8,9], respectively. The bio-distribution of the two kinds of nanoparticles in cells were analyzed by Prussian blue staining. Iron oxide nanoparticles could specially appear blue by Prussian blue staining [27]. In our study, we found that both nanoparticles were similarly absorbed into the cytoplasm; the blue stained nanoparticles were obviously distributed around the nucleus (Figure 2).

### Nanoparticles have no interference on the colorimetric assays for the detection of cell proliferation

In our study, cell viability was determined by colorimetric assay between the cell supernatant and WST-8 kit. Since in previous studies we found that both the glycine- and lysine-coated nanoparticles could adsorb kinds of organic dyes, the possible interference of nanoparticles on the colorimetric assays makes it unavailable to detect the cell proliferation accurately. To evaluate the potential interaction, after color reaction between the cell supernatant with WST-8 kit, 50 μg/ml MNPs@Gly and MNPs@Lys were incubated with cell supernatant at 37°C for 60 min. Then, the optical density (OD) values were observed after the nanoparticles were removed by centrifugation. As shown in Additional file 1: Figure S1, for the cases of normal culture and starvation, both nanoparticles did not interfere the OD values of cell viability. These data indicate that the WST-8 kit is able to detect cell proliferation.

### Cell morphological and viability changes of HUVECs treated with the nanoparticles

**Figure 3:**
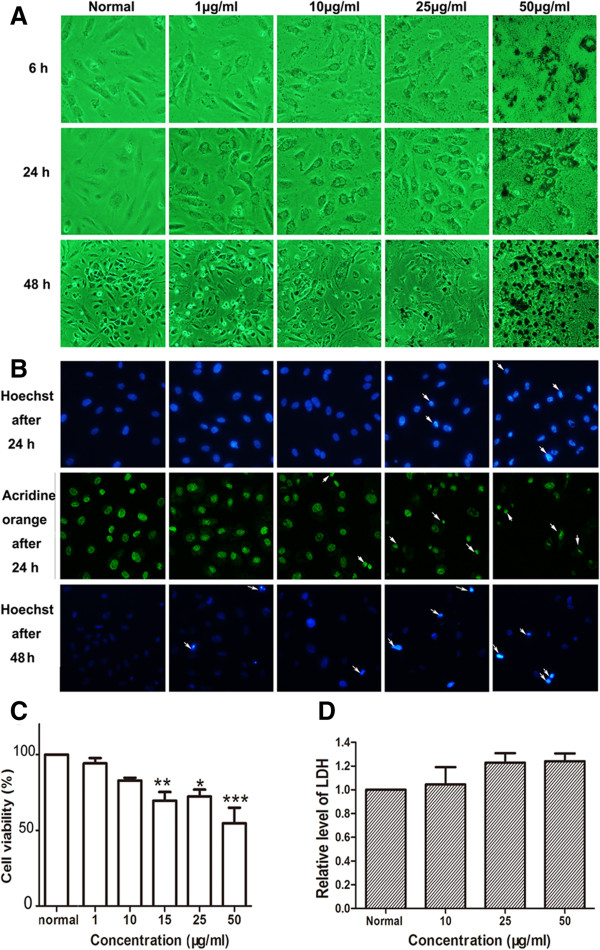
**Effects of MNPs@Gly on HUVECs. (A)** Morphological observation of HUVECs treated with MNPs@Gly for 6 h (×200), 24 h (×200), and 48 h (×100) at different concentrations. **(B)** Hoechst 33258 staining (top) and acridine orange staining (middle) for HUVECs treated with the nanoparticles for 24 h. Hoechst 33258 staining for HUVECs treated with the nanoparticles for 48 h (bottom). Arrows indicated the dead cells. **(C)** WST-8 assay determined the viability of HUVECs treated with the nanoparticles for 48 h at different concentrations (*P < 0.05, **P < 0.01, ***P < 0.001 vs normal, *n* = 3). **(D)** LDH assay detected no cell necrosis. Data were expressed as relative level of LDH compared with normal (*n* = 3).

**Figure 4:**
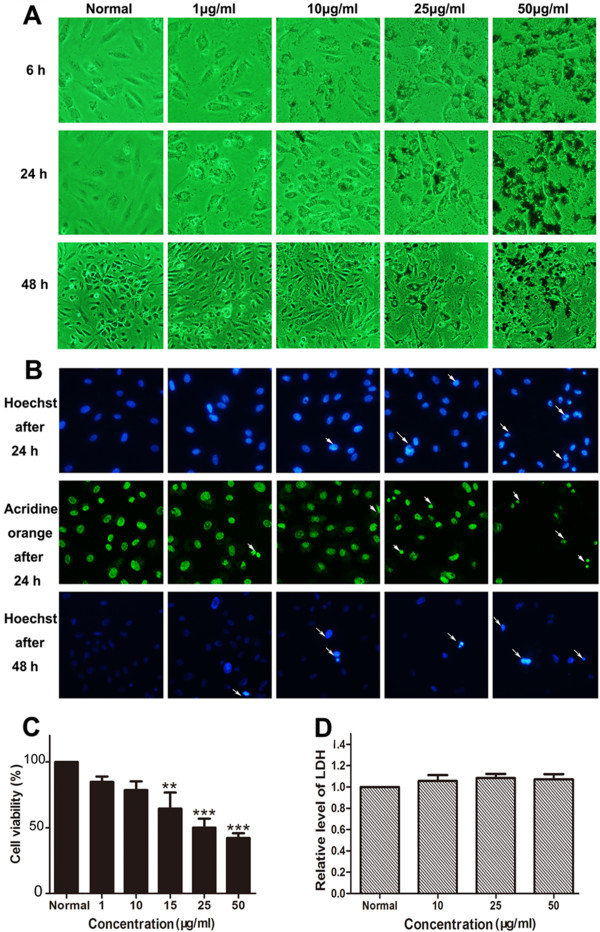
**Effects of MNPs@Lys on HUVECs. (A)** Morphological observation of HUVECs treated with MNPs@Lys. **(B)** Hoechst 33258 staining (top) and acridine orange staining (middle) after 24 h. Hoechst 33258 staining after 48 h (bottom). Arrows indicated the dead cells. **(C)** WST-8 assay determined the viability of HUVECs treated with the nanoparticles for 48 h at different concentrations (**P < 0.01, ***P < 0.001 vs normal, *n* = 3). **(D)** LDH assay detected no cell necrosis (*n* = 3).

Vascular endothelial cells were the first biological barriers when nanoparticles were phagocytized through the blood vessels [18]. In this study, we used HUVECs to mimic the nanoparticle-endothelial interactions. To determine the toxic concentration of the two kinds of nanoparticles in HUVECs, the cells were incubated with the two nanoparticles at different concentrations (from 1 to 100 μg/ml) for 6, 24, and 48 h, respectively. The images showed that both MNPs@GLy and MNPs@Lys were distinctly absorbed into cells and dispersed in the cytoplasm as early as 6 h. We clearly found that the nanoparticles accumulated at 50 μg/ml. Moreover, both of them were further swallowed after being incubated for 24 and 48 h. The images also indicated that the nanoparticles induced increasing cell death in a concentration-dependent manner but with no disturbance of cell membrane integrity (Figures 3A and 4A). This was consistent with the results of Hoechst 33258 and AO staining which showed relatively more nucleus condensation or fragments at relatively high concentration (25 and 50 μg/ml) (Figures 3B and 4B). WST-8 assay indicated that both glycine- and lysine-coated nanoparticles significantly reduced cell viability at 15 μg/ml (Figures 3C and 4C).

### Effects of the nanoparticles on morphology and viability of BMSCs

**Figure 5:**
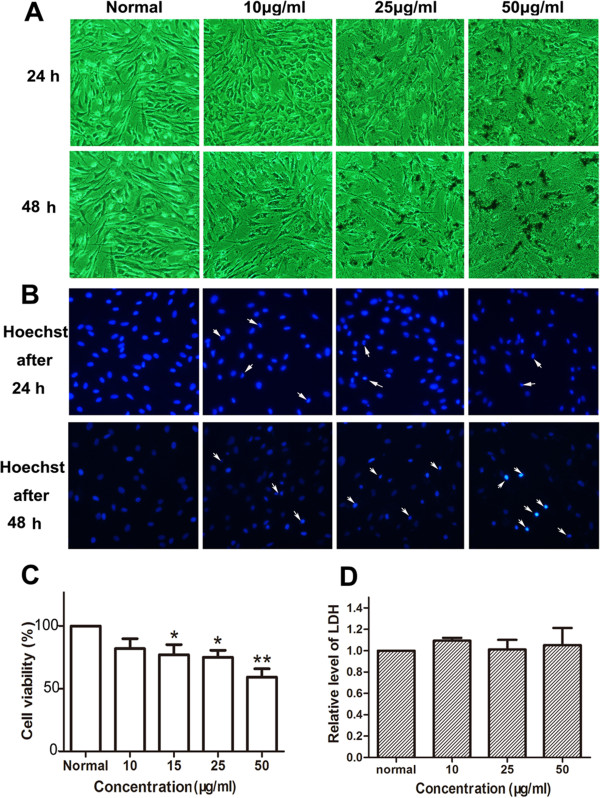
**Effects of MNPs@Gly on BMSCs. (A)** Morphological changes of BMSCs were observed under a phase contrast microscope after the cells were treated with different concentrations of the nanoparticles for 24 h (×100) and 48 h (×100), respectively. **(B)** Changes of the cell nucleus by Hoechst 33258 staining assay after the cells were treated with nanoparticles for 24 and 48 h at different concentrations. **(C)** Cell viability of BMSCs treated with different concentrations of nanoparticles for 48 h by WST-8 assay. These data were presented as percentage compared with the normal group (*P < 0.05, **P < 0.001 vs normal, *n* = 4). **(D)** Relative level of LDH released from BMSCs; the BMSCs were treated with the nanoparticles for 48 h (*n* = 3).

**Figure 6:**
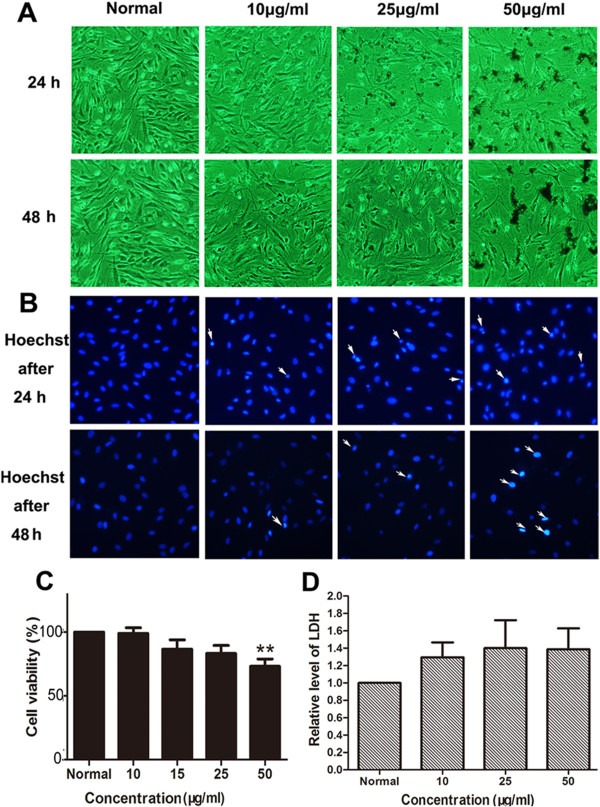
**Effects of MNPs@Lys on BMSCs. (A)** Morphological changes of BMSCs were observed under a phase contrast microscope after treatment with different concentrations of the nanoparticles for 24 h (×100) and 48 h (×100), respectively. **(B)** Changes of the cell nucleus by Hoechst 33258 staining assay after the cells were treated with the nanoparticles for 24 and 48 h at different concentrations. **(C)** Cell viability of BMSCs treated with different concentrations of the nanoparticles for 48 h by WST-8 assay. These data were presented as a percentage compared with the normal group (**P < 0.001 vs normal, *n* = 4). **(D)** Relative level of LDH released from BMSCs; the BMSCs were treated with the nanoparticles for 48 h (*n* = 3).

We used the same methods as above to determine the nanotoxicity in BMSCs. To our surprise, the MNPs were not obviously phagocytized by BMSCs as HUVECs. Moreover, the MNPs obviously resulted in cell loss at higher concentrations (25 and 50 μg/ml) (Figures 5A and 6A). Then, we examined cell apoptosis by Hoechst 33258 staining. We clearly found cell nucleus condensation or fragments after incubation with MNPs at higher concentrations (more than 25 μg/ml) (Figures 4B and 5B). WST-8 assay indicated that MNPs@Gly potently decreased cell viability at 15 μg/ml, while MNPs@Lys decreased cell viability at a concentration of 50 μg/ml (Figures 5C and 6C).

### Effects of the nanoparticles on morphology and viability of THP-1-derived macrophages

**Figure 7:**
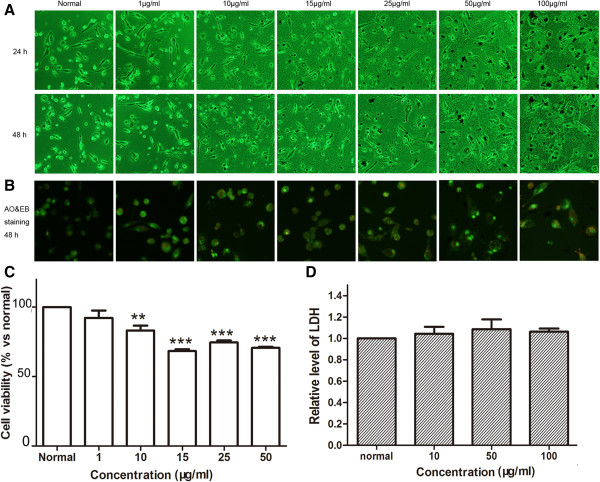
**Effects of MNPs@Gly on macrophages. (A)** Effects of the nanoparticles on cell morphology of THP-1-derived macrophages exposed to the nanoparticles at different concentrations for 24 and 48 h. **(B)** Cell apoptosis or necrosis was determined by AO and EB double staining assay. **(C)** Cell viability was determined by WST-8 assay; the cells were treated with the nanoparticles for 48 h (**P < 0.01, ***P < 0.001 vs normal, *n* = 3). **(D)** Cell necrosis was determined by detecting the release of LDH from THP-1-derived macrophages (the cell density was 40,000/cm^2^, *n* = 3).

**Figure 8:**
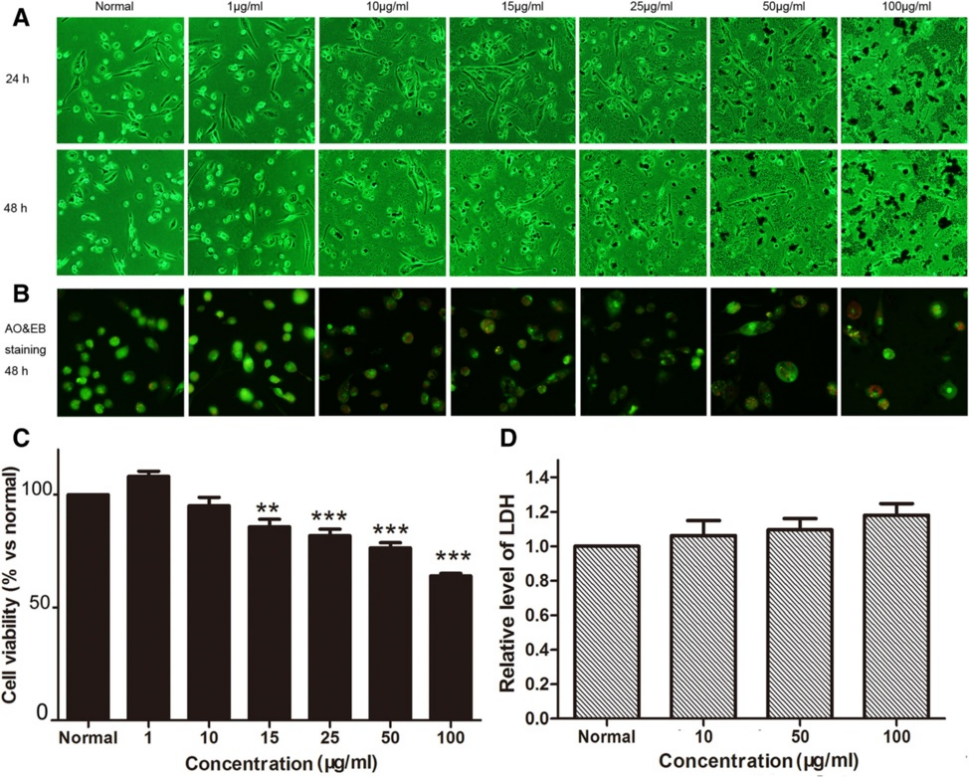
**Effects of MNPs@Lys on macrophages. (A)** Effects of the nanoparticles at different concentrations on cell morphology of THP-1-derived macrophages treated for 24 and 48 h. **(B)** Cell apoptosis or necrosis was determined by AO and EB double staining assay. **(C)** Cell viability was determined by WST-8 assay; the cells were treated with the nanoparticles for 48 h (**P < 0.01, ***P < 0.001 vs normal, *n* = 3). **(D)** Cell necrosis was determined by detecting the release of LDH from THP-1-derived macrophages (the cell density was 40,000/cm^2^, *n* = 3).

To examine the cytotoxicity of the MNPs on macrophages, we initially used PMA to differentiate THP-1 into macrophages. After the exposure of the cells to nanoparticles for 24 or 48 h, we found that the nanoparticles were dramatically swallowed (Figures 7A and 8A). Meanwhile, AO-EB staining indicated that the nanoparticles caused remarkably nucleus condensation. But the red-stained cell nucleus was not found (Figures 7B and 8B), which were regarded as cell necrosis. These results showed that glycine-coated nanoparticles led to dramatic cell loss at 10 μg/ml, while lysine-coated nanoparticles decreased cell viability at 15 μg/ml (Figures 7C and 8C).

### MNPs did not induce cell necrosis

LDH as a cytoplasmic enzyme can be released to the extracellular space and could be detected distinctly in the supernatant when cell membrane integrity is damaged. Therefore, LDH is detected as a typical marker of cell necrosis [35]. We found no difference in LDH release in HUVECs (Figures 3D and 4D), BMSCs (Figures 5D and 6D), and THP-1-derived macrophages (Figures 7D and 8D) after incubation with 50 or 100 μg/ml of nanoparticles for 48 h.

MNPs, owing to their large surface area, high separation efficiency, and low cost [7], have attracted great interest as potential biophysical absorbents. Although more concerns have been raised about the possible detrimental effects of MNPs on human health, the existing information of nanotoxicity on different kinds of cells is still incomplete. In this study, we used HUVECs (as the first and the most important vascular barrier), BMSCs (as one of the most important cells in organizing the hematopoietic system), and macrophages (as a representative of inflammatory response cells) to evaluate the toxicity of two novel amino acid-coated magnetic nanoparticles. Our previous studies reported that the novel negative surface-charged Fe_3_O_4_ magnetic nanoparticles (Fe_3_O_4_@APS@AA-co-CA) could effectively remove heavy metal ions and cationic dyes from aqueous solution [7,36]. The nanoparticles have no influence on the survival of HUVECs until at a high concentration (400 μg/ml). However, the two novel amino acid-coated MNPs (MNPs@GLy and MNPs@Lys), by contrast, have severe damage on HUCECs, BMSCs, and THP-1-derived macrophages, which decreased cell viability at a very low concentration (10 or 15 μg/ml). It has been reported that the nanotoxicity may be size-dependent [37] and surface charge-dependent [38], so big different toxicological implications could be attributed to the contribution of nanoparticle size as well as different surface charge. The diameter of Fe_3_O_4_@APS@AA-co-CA is about 15 to 20 nm, which is larger than the nanoparticles in the present study; a smaller particle size may lead to more serious cytotoxicity [39]. Another important factor may be the different surface charge. These factors may affect the interaction between the nanoparticles and cell membrane, which also leads to the influence of cellular uptake of nanoparticles. Our data further demonstrated that both the nanoparticles were swallowed into cells in a time-dependent and concentration-dependent manner. The nanoparticles were constantly absorbed until the presence of aggregates of nanoparticles.

One important implication of our findings is that the same core nanoparticles coated with different materials may display different nanotoxicity. Biomaterial (e.g., amino acid or peptide)-modified nanoparticles do not necessarily improve the biocompatibility of nanoparticles; these modified nanoparticles, on the contrary, may more easily interact with cell membrane, which contributes to the uptake of nanoparticles and results in greater cytotoxicity. Also, we provide new evidence about the cell type-specific nanotoxicity of amino acid-coated magnetic nanoparticles.

## Conclusions

In summary, we evaluated the potential nanotoxicity of two novel MNPs surface-coated with glycine or lysine in human endothelial cells, macrophages, and rat bone marrow stromal cells. These data suggest that the amino acid-coated magnetic nanoparticles exert relatively high cytotoxicity; lysine-coated magnetic nanoparticles are more secure than glycine-coated magnetic nanoparticles. Therefore, it should be cautious to apply the two nanoparticles in wastewater treatment. Our findings may provide useful information for the further design of iron oxide nanoparticles.

## Abbreviations

AO: acridine orange; BMSCs: bone marrow stromal cells; EB: ethidium bromide; FBS: fetal bovine serum; FGF-2: basic fibroblast growth factor; Gly: glycine; HUVECs: human umbilical vein endothelial cells; LDH: lactate dehydrogenase; Lys: lysine; MNPs: magnetic (Fe_3_O_4_) nanoparticles; MNPs@APS@AA-co-CA: Fe_3_O_4_@3-aminopropyltriethoxysilane@acrylic acid and crotonic acid; MNPs@Gly: Fe_3_O_4_@3-glycidoxypropyltrimethoxysilane@glycine; MNPs@Lys: Fe_3_O_4_@3-glycidoxypropyltrimethoxysilane@Lysine; PMA: phorbol-12-myristate acetate; THP-1: human mononuclear cell line.

## Competing interests

The authors declare that they have no competing interests.

## Authors' contributions

JM, BZ, and QW conceived the study and designed the paper. QW carried out the experiments and wrote the paper. QW and NM co-discussed the results. YZ synthesized the glycine- or lysine-coated MNPs. LH, LS, and JZ discussed and interpreted the results. BZ and SZ prepared the nanoparticles. YZ, BZ, and JM revised the manuscript critically. All authors read and approved the final manuscript.

## Supplementary Material

Additional file 1 **Supplementary information.** This file contains a supplementary table and figure. Click here for file
