# Supplementary material for: The effect of two novel amino acid-coated magnetic nanoparticles on survival in vascular endothelial cells, bone marrow stromal cells, and macrophages
Source: Nanoscale Res Lett. 2014 Sep 3;9(1):461. doi: 10.1186/1556-276X-9-461 (PMC4177720; doi:10.1186/1556-276X-9-461)
Supplement: Additional file 1 — Supplementary information. This file contains a supplementary table and figure. [file 1556-276X-9-461-S1.doc]

# Supplementary information:

# The effect of two novel amino acid-coated magnetic nanoparticles on survival in vascular endothelial cells, bone marrow stromal cells and macrophages

*Qinghua Wu^1^, Ning Meng^2, 3^, Yanru Zhang^2^, Lei Han^1^, Le Su^1^, Jing Zhao^1^, Shangli Zhang^1^, Yun Zhang^4^, Baoxiang Zhao^2^, *, Junying Miao^1, 4^, **

^1^Shandong Provincial Key laboratory of Animal Cells and Developmental Biology, School of Life Science, Shandong University, Jinan 250100, China

^2^Institute of Organic Chemistry, School of Chemistry and Chemical Engineering, Shandong University, Jinan 250100, China

^3^School of Biological Science and Biotechnology, University of Jinan, Jinan 250022, China

^4^ The key laboratory of Cardiovascular Remodeling and Function Research, Chinese Ministry of Education and Chinese Ministry of Health, Shandong University Qilu Hospital, Jinan 250100, China

***Correspondence to**: Prof. Junying Miao and Baoxiang Zhao

Institute of Developmental Biology, School of Life Science, Shandong University,

Jinan, 250100, China

Fax: +8653188565610;

Tel: +8653188364929.

E-mail address: [miaojy@sdu.edu.cn](mailto:miaojy@sdu.edu.cn), and [bxzhao@sdu.edu.cn](mailto:bxzhao@sdu.edu.cn)


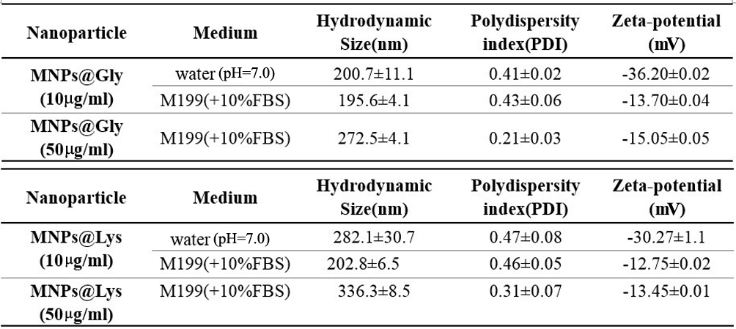


Table S1. Physicochemical characterization of glycine or lysine coated MNPs.


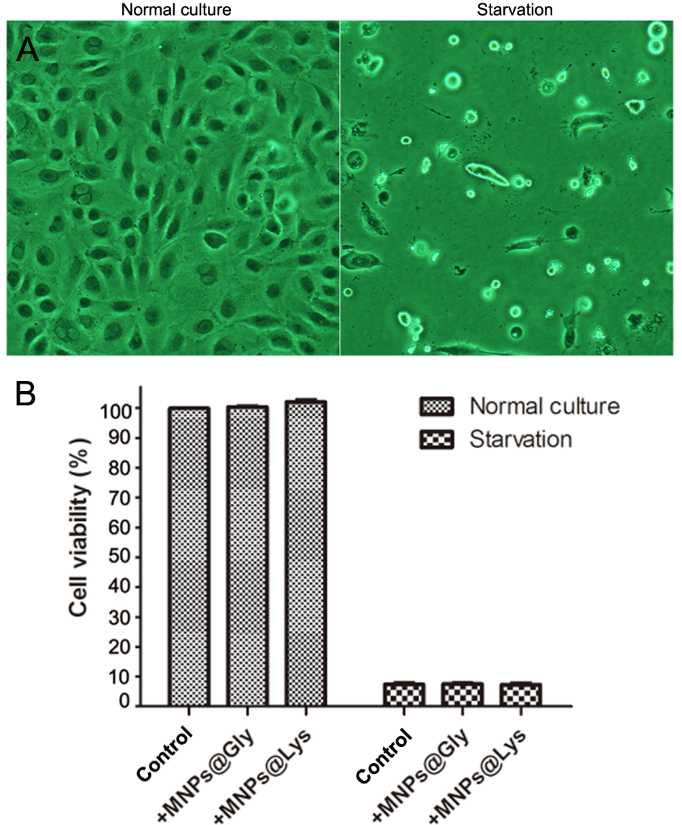


Figure S1. The glycine or lysine coated nanoparticles have no interference on the color metric assay of WST-8 kit. (A) The digital images of HUVECs. Left, cells were cultured with normal culture for 24 h. Right, cells were cultured under starvation condition (serum and FGF-2 free) for 24 h. (B) Cell proliferation was detected by WST-8 kit. Cell supernatant after color reaction incubated with 1×PBS (Control), MNPs@Gly (50 μg/ml) ( +MNPS@Gly) and MNPs@Lys (50 μg/ml) (+MNPs@Lys) for 60 min at 37℃ (vs control, n = 3).
